# Gaps in Guideline-Directed Medical Therapy for Heart Failure: A Cross-Sectional Study in an Underserved Population

**DOI:** 10.7759/cureus.111419

**Published:** 2026-06-24

**Authors:** Ayara O Ehinmisan, Shilpi Jain-Aggarwal, Shweta Karki

**Affiliations:** 1 Cardiology, University Hospitals Cleveland Medical Center, Cleveland, USA; 2 Internal Medicine, Stamford Hospital, Stamford, USA; 3 Research, Stamford Hospital, Stamford, USA

**Keywords:** angiotensin-converting enzyme inhibitors (acei), angiotensin ii receptor blockers, beta blockers, guideline directed medical therapy, hfpef (heart failure with preserved ejection fraction), hfref (heart failure with reduced ejection fraction), mineralocorticoid receptor antagonist, sglt-2 inhibitor

## Abstract

Background: Heart failure (HF) remains a major clinical and public health challenge. Guideline-directed medical therapy (GDMT) has been shown to improve outcomes in patients with HF; however, gaps in the prescription and implementation of these therapies persist. These treatment gaps may contribute to suboptimal disease management and adverse clinical outcomes, highlighting the need to better understand patterns of GDMT utilization in diverse patient populations. In this study, we assessed and evaluated the prescribing patterns of GDMTs to an underserved population at an urban federally qualified health center (FQHC).

Methods: We conducted a retrospective chart review of patients diagnosed with HF from May 2022 to June 2024 to assess the prescription of GDMT. Patients were classified based on echocardiographic findings as HF with reduced ejection fraction (HFrEF) (< 50%) and HF with preserved ejection fraction (HFpEF) (≥ 50%). Baseline demographics and prescription of GDMTs were compared between the two HF diagnoses.

Results: Fifty patients with HF were identified in the study period, including 41 with HFrEF and nine with HFpEF. There was a significant difference in the mean age between patients diagnosed with HFpEF (68 years) as compared to patients with HFrEF (58 years) (p=0.02). Among patients with HFrEF, 93% (n=38) were prescribed beta-blockers (BB), 83% (n=34) were prescribed angiotensin-converting enzyme inhibitors/angiotensin II receptor blockers/angiotensin receptor-neprilysin inhibitors (ACEi/ARB/ARNi), 29% (n=12) were prescribed mineralocorticoid receptor antagonists (MRA), and 34% (n=14) were prescribed sodium-glucose cotransporter-2 inhibitors (SGLT2). Prescriptions for these medical therapies were similar among patients with HFpEF, except that 0% were prescribed an SGLT2 medication (p=0.05 for comparison with patients with HFrEF).

Conclusions: GDMT is under-prescribed in underserved populations, underscoring the need for specific interventions to address financial, systemic, and educational barriers. Interventions such as patient education initiatives, financial counseling support, provider training programs, and policy changes to improve medication affordability and access should be prioritized to help close these treatment gaps.

## Introduction

Guideline-directed medical therapy (GDMT) encompasses a class of pharmacological agents, including beta-blockers (BB), angiotensin-converting enzyme inhibitors/angiotensin II receptor blockers/angiotensin receptor-neprilysin inhibitors (ACEi/ARB/ARNi), mineralocorticoid receptor antagonists (MRA), sodium-glucose cotransporter-2 inhibitors (SGLT2), and vasodilators. These agents constitute the cornerstone of pharmacotherapy for heart failure with reduced ejection fraction (HFrEF) [1].

The most recent guidelines from the American Heart Association, the Heart Failure Society of America (HFSA), and the American College of Cardiology recommend these medications as Class I for concurrent use in HFrEF management, with evidence of mortality reduction [2]. Notwithstanding their demonstrated efficacy, research indicates that many patients do not receive all four pillars of GDMT, even as the prevalence of HF escalates [3]. Although novel medications and technologies continue to emerge, the morbidity and mortality burden of HF persists substantially due to ongoing gaps in the implementation of therapeutic advancements [4]. Diverse barriers underlie the underprescription of these therapies.

While GDMT underutilization has been documented in hospital and specialty care settings, data on prescribing patterns specifically within federally qualified health centers (FQHCs) serving underserved, low-income, and uninsured populations remain limited. In this investigation, we assessed GDMT prescription in a primary care outpatient setting at an FQHC. This article was previously presented as a meeting abstract at the 2025 HFSA annual scientific meeting on September 27, 2025.

The primary objective of this study was to quantify GDMT prescription rates among patients with HF receiving care at an urban FQHC serving a predominantly low-income and uninsured population. Secondary objectives were to compare GDMT prescribing patterns between patients with HFrEF and HF with preserved ejection fraction (HFpEF) and to identify potential barriers to under-prescription in this setting.

## Materials and methods

Study design and population

The study was conducted at the Optimus Clinic, Stamford, CT, USA. This cross-sectional study included patients with a diagnosis of HF documented in the Epic electronic medical record who had ≥1 clinic visit between May 1, 2022, and May 31, 2024. Patients were identified by an independent statistician using International Classification of Diseases, Tenth Revision (ICD-10) codes I50.20, I50.21, I50.22, I50.23, I50.30, I50.31, I50.32, and I50.33 in the Epic electronic medical record system [5]. All extracted charts were subsequently cross-checked by the first author to verify the documented diagnosis of HF and to confirm that available echocardiographic data correlated with the assigned HF phenotype. For the purposes of this study, a medication was considered prescribed if it was ever ordered for the patient at any point during the study period as documented in the Epic electronic medical record. A retrospective chart review was performed to confirm HF diagnosis and classify phenotype based on left ventricular ejection fraction (LVEF) obtained from echocardiography. Race included White, Black, and Other patients (Others included American Indian, Asian, Native Hawaiian, and more than one race). Western Institutional Review Board (WIRB)-Copernicus Group issued approval 1-1811589-1 for this study.

Table 1 summarizes the categorization of HF phenotypes according to ejection fraction used in our study. Although the most recent guidelines from the American Heart Association, the HFSA, and the American College of Cardiology distinguish three HF phenotypes: HFrEF (LVEF ≤40%), HF with moderately reduced ejection fraction (HFmrEF) (LVEF 41-49%), and HFpEF (LVEF ≥50%), we combined HFrEF and HFmrEF into a single group (LVEF <50%) given the small overall sample size and the clinical overlap in GDMT recommendations across these two phenotypes, where the same drug classes carry Class I indications for HFrEF and Class IIa/IIb indications for HFmrEF.

**Table 1:** Heart failure phenotypes based on left ventricular ejection fraction HFrEF: heart failure with reduced ejection fraction; HFpEF: heart failure with preserved ejection fraction

Definitions	Ejection Fraction
HFrEF	EF <50%
HFpEF	EF ≥50%

Table 2 shows our inclusion and exclusion criteria. Patients were enrolled in our study based on the criteria listed in our table.

**Table 2:** Inclusion and exclusion criteria HF: heart failure

Inclusion Criteria	Exclusion Criteria
1. Age 18–80 years	1. Advanced HF therapies (cardiac transplantation or left ventricular assist device)
2. Confirmed diagnosis of HF with available echocardiographic data	2. Missing echocardiographic data or studies performed >1 year prior to the study period
	3. Congenital heart disease

Statistical analysis

A priori power calculations were not feasible due to the absence of established effect sizes. Post hoc power analyses were performed using independent tests of proportions (two-sided α=0.05) with power estimates of 80% and 90%.

Data were analyzed using SAS version 9.4 (SAS Inc., Cary, NC, USA). Categorical variables are presented as frequencies and percentages; continuous variables are expressed as mean ± SD. Comparisons between HF phenotypes were performed using chi-square or Fisher's exact tests, as appropriate.

## Results

Table 3 shows the baseline demographics of our patients, which include their ethnicity, race, insurance status, and age. There was a statistically significant difference in age among patients with HF (p=0.02). The mean age of patients with pEF was higher (68 years) compared to patients with rEF (58 years).

**Table 3:** Baseline demographics

Variables	Category	Heart Failure Diagnosis		p-value
		Preserved ejection fraction (pEF) n=9	Reduced ejection fraction (rEF) n=41	
Ethnicity	Hispanic	5 (62.5%)	19 (46.3%)	0.33
	Not Hispanic	3 (37.5%)	21 (51.22%)	
	Unknown	1 (11.1%)	1 (2.44%)	
Race	White	3 (33.3%)	14 (34.2%)	0.99
	Black	3 (33.3%)	11 (26.83%)	
	Other	3 (33.3%)	16 (39.02%)	
Insurance	Medicare/Medicaid	3 (33.3%)	11 (26.8%)	0.69
	Self-pay	6 (66.7%)	30 (73.2%)	
Age	Years	68.37 (10.87)	58.24 (11.37)	0.02

Table 4 presents the distribution of GDMT across HF subtypes. Medication prescribing patterns were compared between patients with pEF (n=9) and rEF (n=41) across four drug classes: SGLT2 inhibitors, BB, ACE inhibitors/ARBs/ARNis, and MRAs.

**Table 4:** Assessing prescriptions for patients diagnosed with heart failure (HF) SGLT2: sodium-glucose cotransporter 2 inhibitors; BB: beta blockers; ACEi/ARB/ARNi: angiotensin-converting enzyme inhibitors/angiotensin II receptor blockers/angiotensin receptor-neprilysin inhibitors; MRA: mineralocorticoid receptor antagonists Fisher's exact test was used due to small expected cell counts. Cramér's V is reported as the effect size.

Variables	Category	Heart Failure Diagnosis		p-value	χ²(df=1)	Cramér's V
		Preserved ejection fraction (pEF) n=9	Reduced ejection fraction (rEF) n=41			
SGLT2	Yes	0	14 (34%)	0.05	4.27	0.29
BB	Yes	8 (89%)	38 (93%)	0.56	0.14	0.05
ACEi/ARB/ARNi	Yes	6 (67%)	34 (83%)	0.36	1.22	0.16
MRA	Yes	2 (22%)	12 (29%)	0.99	0.18	0.06

There was a significant difference in SGLT2 inhibitor prescription among patients with HF. Among patients with rEF, 66% (n=27) were not prescribed an SGLT2 inhibitor, whereas 100% n=9 of patients with pEF were not prescribed (p=0.05). 

Although lower prescription rates were observed in patients with rEF compared to those with pEF, these differences were not statistically significant. Among patients with rEF, 93% (n=38) were prescribed, and 7% (n=3) were not prescribed BB. Around 83% (n=34) were prescribed ACEi/ARB/ARNi, 17% (n=7) were not prescribed ACEi/ARB/ARNi, and 29% (n=12) were prescribed, and 71% (n=29) were not prescribed MRAs. 

## Discussion

FQHCs are ambulatory clinics that provide primary care to underserved, low-income, or uninsured populations regardless of their ability to pay [6]. Among these patients, affordability may represent a potential barrier to GDMT, even as many agents have become generics [7].

For example, as of 2018, the annual Medicare costs for carvedilol, lisinopril, and spironolactone averaged $173, including approximately $64 in out-of-pocket expenses. Although 90-day supplies can cost as little as $10 in some pharmacies, these prices may still be prohibitive for very low-income individuals. In contrast, newer therapies such as sacubitril-valsartan and dapagliflozin carry annual costs exceeding $10,000, with insured patients facing substantial out-of-pocket costs and uninsured patients facing even greater financial burden. Given that approximately 72% of our study population was uninsured, these barriers are likely to be particularly important in our cohort [8].

In addition to cost, limited health literacy and mistrust of the healthcare system may represent additional potential barriers among underserved populations. This mistrust is especially pronounced in African American communities because of historical injustices in medical research and medical care delivery, resulting in reduced engagement in both clinical care and research participation [9]. Socioeconomic constraints may also limit access to specialists and multidisciplinary teams that optimize HF therapy [10].

From a system perspective, workforce diversity and culturally responsive care remain important components of reducing healthcare mistrust and increasing patient engagement. Increasing the representation of physicians from underrepresented backgrounds has been associated with improved communication, trust, and observation among historically marginalized populations [3]. Continuing obstacles to diversity, equity, and inclusion initiatives may further widen disparities in access to medical care and outcomes, notably in vulnerable communities [11,12].

To our knowledge, this is one of the few studies to evaluate GDMT prescribing patterns specifically within an FQHC serving a predominantly low-income and uninsured urban population, filling an important gap in the existing literature. Our findings are consistent with those from larger registries. The CHAMP-HF (Change the Management of Patients with HF) study demonstrated that 27% of patients with HFrEF were not prescribed ACEi/ARB/ARNi therapy, 33% were not receiving BB, and 67% were not receiving MRAs [13]. SGLT2 inhibitors, despite their incorporation into contemporary guidelines on HF, continue to show suboptimal prescribing rates in eligible patients [14]. It is also worth noting that SGLT2 inhibitors were formally incorporated into HF Guidelines in 2022, coinciding with the start of our study period, and the low prescribing rates observed may partly reflect the early adoption phase of these agents in routine clinical practice; however, broader literature has consistently demonstrated that SGLT2 inhibitors remain underutilized even beyond the initial period of guideline incorporation in our cohort. In our cohort, MRAs and SGLT2 inhibitors represented the least prescribed components of GDMT. Unlike large national registries, which predominantly capture patients managed in hospital and specialty care settings, our study uniquely characterizes GDMT prescribing in a primary care outpatient FQHC, where patients are predominantly low-income, uninsured, and ethnically diverse, a population that remains largely underrepresented in the existing HF literature.

We included patients with HFpEF despite the small sample size because SGLT2 inhibitors now carry a Class IIa recommendation for this phenotype [2]. Notably, none of the HFpEF patients in our cohort were prescribed SGLT2 inhibitors. Collectively, these findings show the persistent underutilization of GDMT, especially among marginalized and resource-limited populations.

It is important to note that our cross-sectional design cannot identify the reasons for under-prescribing or predict which interventions will be most effective; the proposed strategies discussed below are therefore informed by the broader HF literature rather than conclusions directly supported by our dataset. The persistent gap between guideline recommendations and real-world prescribing patterns underscores the need for interventions at multiple levels [15,16]. Potential strategies include policy-level efforts to improve medication affordability and insurance coverage, expanded provider education in ambulatory settings, and the incorporation of multidisciplinary care teams, including pharmacists and social workers [17]. In addition, patient-centered educational initiatives and financial counseling may help mitigate barriers related to medication costs and health literacy [18]. Several studies have demonstrated that these interventions can be effective in increasing GDMT uptake. Pharmacist-led HF management programs have been associated with marked improvement in GDMT prescription rates and patient adherence. For example, a pharmacist-led virtual ward uptitration program achieved prescription of all four foundational therapies in 84% of patients and demonstrated meaningful improvement in ejection fraction [19]. The economic and clinical benefits of pharmacist integration in ambulatory HF clinics have been further confirmed through a scoping review of evidence [20]. Multidisciplinary clinics have similarly demonstrated improved medication optimization and clinical outcomes [21]. Patient financial obstacles are a critical driver of nonadherence, and pharmacist-led programs consistently incorporate co-pay assistance and patient assistance program navigation to enable guideline-based care [22]. Future research needs to focus on developing and evaluating integrated care models that improve coordination between cardiologists, primary care providers, pharmacists, advanced practice providers, and patients to improve GDMT adherence and optimization [23].

There are several limitations to our study. First, the small sample size, particularly in the pEF group (n=9), limits the analyses' statistical power and may have contributed to the inability to detect significant differences across most medication variables. The use of Fisher's exact test, required by expected cell counts below five in multiple comparisons, reflects these sample size constraints. Second, the considerable imbalance between groups (n=9 pEF vs. n=41 rEF) may introduce selection bias and limit the generalisability of the findings. Third, while a borderline significant difference in SGLT2 inhibitor use between groups was observed (V=.29, p=.047), the small-to-medium effect size should be interpreted cautiously given the limited sample. Fourth, the negligible effect sizes observed for BB (V=.05), ACEi/ARB/ARNI (V=.16), and MRA (V=.06) use may reflect true clinical parity between groups, or alternatively may be a consequence of insufficient statistical power to detect meaningful differences. Fifth, as a cross-sectional analysis of medication prescribing patterns, this study cannot demonstrate causality or account for potential confounders such as disease severity, comorbidities, or physician prescribing practices that may influence medication selection across subtypes of HF. Sixth, our study did not systematically assess for contraindications to GDMT, including medication intolerance, renal dysfunction, hyperkalemia, or hypotension, which may have clinically precluded prescribing in some patients, and we were therefore unable to report prescription rates among patients eligible for each individual GDMT class; as a result, the observed rates of under-prescription should be interpreted cautiously, as they may overestimate the true gap in guideline-directed therapy. Seventh, as this was a single-center study conducted at one urban FQHC, the findings may not be generalizable to other FQHC settings or HF populations. Eighth, our study assessed whether medications were prescribed during the study period, but did not evaluate whether they were prescribed at guideline-recommended target doses or whether patients were adherent to their prescribed regimens, as prescribed medications do not necessarily equate to medications taken. Finally, our retrospective chart review did not capture the reason why specific medications were not prescribed, precluding differentiation between physician choice, patient refusal, and clinical contraindications as drivers of under-prescription.

## Conclusions

Our findings provide local implementation evidence consistent with the broader literature, suggesting that GDMT remains substantially underutilized among underserved patients receiving care at an FGHC, with particularly low prescribing rates observed for MRAs and SGLT2 inhibitors. Financial barriers, lack of insurance coverage, limited healthcare access, and broader social determinants of health may contribute to these disparities; however, because these variables were not directly measured in our study, they remain plausible hypotheses warranting further investigation.

While limited by its small, single-center, cross-sectional design, our study underscores the need for multifaceted interventions to improve GDMT utilization in vulnerable populations. Expanding low-cost medication programs and strengthening coordinated HF management strategies may help bridge the gap between guideline recommendations and real-world implementation in resource-limited settings.
